# Plasma heparanase is associated with blood glucose levels but not urinary microalbumin excretion in type 2 diabetic nephropathy at the early stage

**DOI:** 10.1080/0886022X.2017.1384391

**Published:** 2017-10-10

**Authors:** Yue Zhao, Jingshun Liu, Shichao Ten, Jisheng Zhang, Yanggang Yuan, Jiangyi Yu, Xiaofei An

**Affiliations:** a Department of Endocrinology, Jiangsu Province Hospital of Chinese Medicine, Affiliated Hospital of Nanjing University of Chinese Medicine, Nanjing, Jiangsu, China;; b Department of Otorhinolaryngology, Affiliated Hospital of Qingdao University, Qingdao, Shandong, China;; c Department of Nephrology, The First Affiliated Hospital of Nanjing Medical University, Jiangsu Province People’s Hospital, Nanjing, Jiangsu, China

**Keywords:** Diabetic nephropathy, heparanase, albuminuria

## Abstract

**Aim:** To explore the possible correlations between plasma heparanase and albuminuria, glucose and lipid metabolism in the type 2 diabetic nephropathy patients at the early stage.

**Methods:** One hundred and forty patients with type 2 diabetic nephropathy at early stage were recruited into the study. Plasma heparanase and the characterized advanced glycation end products (AGEs), carboxymethyllysine (CML) were measured by enzyme-linked immunosorbent assay.

**Results:** Plasma heparanase was positively associated with fasting blood glucose (*R* = 0.24, *p* = .01) while heparanase was not significantly correlated with the urinary microalbumin to creatinine ratio (urinary mAlb/Cr) (*R* = 0.05, *p* = .58) and CML (*R* = 0.16, *p* = .26). On stepwise linear regression analysis, fasting blood glucose was the main independent determinants of plasma heparanase concentration.

**Conclusion:** Plasma heparanase is not significantly associated with urinary mAlb/Cr while it is correlated positively with blood glucose levels in the early stage of diabetic nephropathy. Plasma heparnase might be regarded as a marker for vascular endothelial cells injury in diabetic patients.

## Introduction

Diabetic nephropathy (DN) is one of the diabetic micro-vascular complications and listed as one of leading cause of end-stage renal disease (ESRD) [1]. Persistent micro-albuminuria is regarded as not only the diagnostic criteria of the early stage of DN [2], but also the risk factor of development of renal injury in DN [3,4]. Although many theories are adopted to explain the onset and development of proteinuria in patients with DN [5,6], the accurate molecular and cellular mechanisms remain to be elucidate till now.

Heparanase is an endo-β-d-glycosidase that specially degrades heparan sulfate which is the main component of heparan sulfate proteoglycans (HSPGs) [7]. Heparanase is associated with cell adhesion, matrix metabolism, tumor metastasis and angiogenesis [8,9]. The glomerular basement membrane (GBM) contains HSPGs as the structural components and the role of HSPGs in GBM has been regarded as the key molecules responsible for the charge-selective characteristic of glomerular filtration barrier [10]. The GBM selective permeability for negatively charged molecules such as albumin is destroyed due to HSPGs degrading by glomerular heparanase [11].

The over-expression of heparanase was observed in several glomerular diseases [12] and glomerular heparanase up-regulation play a pivotal role in the development of DN [13,14]. In diabetic status, over-expression of heparanase could be induced by high glucose [15,16] and advanced glycation end products (AGEs) [17,18]. The levels of glomerular heparanase expression were found to be strongly associated with the urinary microalbumin excretion in DN animal model or DN patients [13,19]. The recent studies showed that increased plasma heparanase levels were found in type 2 diabetic patients and urine heparanase was associated with elevated blood glucose levels [20]. Urinary heparanase was markedly elevated in kidney-transplanted patients and urinary heparanase was markedly elevated and associated with proteinuria in chronic kidney disease (CKD) patients [21]. Urinary heparanase is found to be significantly correlated with plasma heparanase in kidney-transplanted patients [21]. These accumulating studies raise the possibility that plasma heparanase could be an early predicting marker for proteinuria in DN patients. The present study aimed to investigate whether plasma heparanase is associated with the levels of micro-albuminuria and related metabolic indexes in the type 2 DN patients at the early stage.

## Methods

One hundred and forty type 2 diabetic patients were recruited from our diabetes outpatient clinic for specialist treatment after giving written consent. The program was supported by the Great Project of National Scientific Supporting Program of China and approved by the ethics committee of Jiangsu Province Hospital of Chinese Medicine (Approval No. 2007NL008). The patients had required testing positive for albuminuria in three separate spot urine samples (30–300 mg/24 h) within 6 months period as our previous study [22]. The blood pressure and the ratio of urinary microalbumin to creatinine (mAlb/Cr) were measured on three consecutive days before study starting. The patients with diagnosed macrovascular complications, poly-neuropathy, proliferative retinopathy and systematic disease (such as cancer, severe infection, AIDS, mental disease) were excluded. To avoid the possible influence on urinary albumin excretion, the patients who got the treatment of the angiotensin-converting enzyme inhibitor (ACEI), angiotensin receptor antagonist (ARB) therapy, statins or antiplatelet drugs were not permitted to recruitment. All the subjects underwent a complete history and physical examination, determination of blood chemistries and their basic characteristics were collected.

Plasma heparanase and the characterized AGE carboxymethyllysine (CML) were measured by enzyme-linked immunosorbent assay (USCN Life Science &Technology Company, Double Lake, MO and R&D Systems, Wiesbaden, Germany, respectively) according to operating instructions [22]. Values were expressed as mean ± standard deviation (SD). For statistical evaluation, variables were correlated by Pearson’s coefficient. When relative Pearson’s correlation coefficients were positive, a backward stepwise multivariate linear regression model was adopted to analyze independent associations of variables with plasma heparanase levels and urinary mAlb/Cr. The *F*-value for the inclusion and exclusion of variables was set at 2.0. A probability of *p* < .05 was considered to be statistically significant. SPSS 16.0 software was used for all statistical analysis (SPSS Inc., Chicago, IL).

## Results

Plasma heparanase levels in the recruited patients with type 2 DN at early stage (326.9 ± 57.2 pg/ml) were similar to the concentrations previously detected in CKD patients (136.9 ± 24 pg/ml) [21] using the same assay. As shown in Table 1, plasma heparanase was positively associated with the level of fasting glucose (*R* = 0.24, *p* = .01) in our studies. There was no significant correlation of mean systolic [*R* = −0.08, *p* = NS (no significance)] or diastolic (*R* = −0.01, *p* = NS) blood pressure with plasma heparanase. Heparanase also did not correlate with CML (*R* = 0.16, *p* = NS), HbA1c (*R* = 0.02, *p* = NS), body mass index (BMI) (*R* = −0.09, *p* = NS) or plasma lipids. Furthermore, heparanase was not significantly correlated with the urinary mAlb/Cr (*R* = 0.05, *p* = NS).

**Table 1.** Correlation between plasma heparanase and variables in all subjects (*n* = 140).

		Univariate		Mutivariate	
Characteristics	Values	R	*p*	R	*p*
Age (years)	61.4 ± 6.5	−0.12	NS	–	–
Sex (male/female)	72/68	−0.08	NS	–	–
Diabetes durations (years)	6.1 ± 4.5	−0.12	NS	–	–
BMI (kg/m^2^)	24.1 ± 2.8	−0.09	NS	–	–
FBG (mmol/L)	7.1 ± 2.0	0.24	.01	0.26	.01
HbA1c (%)	6.5 ± 1.3	0.02	NS	–	–
DBP (mmHg)	76.4 ± 9.0	−0.01	NS	–	–
SBP (mmHg)	129.6 ± 13.9	−0.08	NS	–	–
Total cholesterol (mmol/L)	4.8 ± 0.9	−0.08	NS	–	–
Triglyceride (mmol/L)	1.5 ± 1.0	−0.03	NS	–	–
HDL (mmol/L)	1.3 ± 0.3	−0.04	NS	–	–
LDL (mmol/L)	2.9 ± 0.6	0.03	NS	–	–
Urinary mAlb/Cr (mg/g)	34.8 ± 15.3	0.05	NS	–	–
CML (ng/ml)	46.5 ± 11.2	0.16	NS	–	–

Data are given as mean ± standard deviation (SD). NS means no significance.

In a stepwise linear regression model, plasma heparanase is independently correlated with fasting glucose level (*b* = 0.28, *p* = .01, *R*^2^ = 0.42). There were no significant differences in heparanase levels between the patients with different urinary mAlb/Cr (*R* = 0.13, *p* = NS). Furthermore, the results of the linear regression model did not change when sex, diabetic duration, blood pressure, plasma lipids and other basic characteristics were added in a larger regression model.

## Discussion

DN is one of the most common and severe micro-vascular complications and affects approximately one-third diabetes mellitus patients worldwide [1]. Albuminuria is characterized clinically as a measure of the severity and determinant for DN. Micro-albuminuria is the predominant renal risk factor in patients with type 2 DN, as well as a target for therapy [3]. Multiple mechanisms are involved in the onset and development of albuminuria in DN, such as high glucose and lipids toxicity, hemodynamic abnormality, oxidative stress, AGEs accumulation, sorbitol formation and immunologic injury [5,6]. The detailed mechanisms and early predictor for progression of DN remain to be investigated.

As an endo-β-d-glycosidase which cleaves heparan sulfate (HS) side chains, heparanase is involved in cellular matrix metabolism associated with tumor metastasis, inflammation and angiogenesis [12,23]. HS act as the major ingredient of GBM [11] and its degradation was observed in experimental DN animal model and human DN [19,24]. Emerging evidence indicated that glomerular heparanase is engaged in several renal diseases primarily in DN [12] and the levels of albuminuria is significantly associated with the heparanase over-expression in DN [13]. With the use of heparanase knockout mice, it was observed that the deletion of the heparanase gene protects diabetic mice from DN [25]. The specific heparanase inhibitor could markedly decrease the extent of albuminuria and renal damages in mouse models of DN [25]. Under diabetic conditions, heparanase over-expression in glomerular cells induces activation of macrophage-mediated renal injury and creating chronic inflammatory conditions [26]. Those studies mentioned above indicated that glomerular heparanase plays a pivotal role in pathogenesis of DN. The recent study showed that urinary heparanase was notably increased and associated with the levels of proteinuria in CKD patients and urinary heparanase is also significantly correlated with plasma heparanase [21]. Increased heparanase levels were also found in the plasma of type 2 diabetic patients [20]. There was hypothesis that plasma heparanase was closely related with the level of urinary albumin excretion and could be taken as a clinical predictor for early-stage DN.

Unexpectedly, our present study suggested that plasma heparanase is not significantly associated with urinary mAlb/Cr while it is correlated positively with fasting blood glucose in a cohort of subjects with early-stage DN. Screening for microalbuminuria in our research has been performed by the measurement of the urinary mAlb/Cr in random spot collection in three consecutive days. It is more convenient and accurate than 24 h urine collection and is the preferred method of American Diabetes Association [2]. The insignificant association between plasma heparanase and urinary mAlb/Cr in our study suggests that heparanase acting as a potential biological marker and predictor for the early stage of type 2 DN still needs more support of further clinical investigation. Because in our cohort, the severity of albuminuria in early-stage DN patients is comparatively lower (urinary mAlb/Cr is ∼34.8 ± 15.3 mg/g), it is uncertain if plasma heparanase is significantly associated with in advanced DN patients.

The previous study showed that both urine and plasma heparanase were significantly associated with elevated blood glucose levels in a cohort of 29 diabetic patients [20]. Remarkably, the diabetic complications of those subjects in their cohort was not detailed introduced, especially their albuminuria levels. Comparatively, our cohort including 140 early-stage type 2 DN subjects was bigger and more homogeneous. Although consistent with previous study [20], our research further suggested that there was an independent positive correlation of plasma heparanase with blood glucose levels (fasting glucose), the data in our observation was more solid and persuasive than that in the previous study. It was reported that high glucose could induce heparanase expression in endothelial cells and glomerular podocyte [15,16]. Plasma heparanse come from up-regulated expression and secretion of heparanase of vascular system especially endothelial cells to circulation [7]. The variation of plasma heparanse is accompanied with the expression and release of heparanase endothelial cells which might be vulnerable to transient and subtle changes of blood glucose such as fasting glucose. Hyperglycemia was considered as the most important factor inducing heparanse expression and secretion. The independent correlation between plasma heparanase and fasting glucose indicted that plasma heparanase could be regarded as sensitive marker of vascular endothelial cells injury induced by high blood sugar in diabetic patients. Due to the inconvenience of urine collection and difficulty in urine heparanase assay, plasma heparanase could be accepted as a better index for high glucose-related vascular injury in DN patients.

Diabetic condition is specially characterized with high glucose and AGEs accumulation and AGEs level is also associated with HbA1c and chronic-elevated blood sugar. AGEs treatment could also increase heparanase expression in endothelial cells and macrophage through its receptor RAGE [17,18]. In the present study, plasma heparanase was not found to be associated with the characterized AGEs, CML and HbA1c, implying that plasma heparanase variation is not due to impact of chronic high glucose. Meanwhile, since there was no significant correlation of plasma heparanase with blood pressure, BMI and plasma lipids levels, hemodynamics and lipids metabolism dysfunction could be excluded as an underlying factor affecting plasma heparanase in diabetic patients.

In summary, the present study demonstrated that plasma heparanase is not significantly associated with urinary microalbumin excretion but is positively associated with blood glucose in early stage of type 2 DN. Plasma heparnase might be regarded as a marker for vascular endothelial cells injury in diabetic patients. Its predicting role in development of microalbuminuria and diabetic glomerular injury needs further prospective clinical investigations.
